# Supplementary material for: Efficient deep learning model for classifying lung cancer images using normalized stain agnostic feature method and FastAI-2
Source: PeerJ Comput Sci. 2025 May 27;11:e2903. doi: 10.7717/peerj-cs.2903 (PMC12192963; doi:10.7717/peerj-cs.2903)
Supplement: Supplemental Information 2 [file peerj-cs-11-2903-s002.docx]

**Procedure to run the application**

1. Load the data (LC25000), please follow the same directory structure as shared.
2. Performed data augmentation and color transformation with colortransformation.ipynb file, followed by post processing.
3. After normalization process, classification framework is established using FastAI-2, and optimal hyperparameters are stored.
4. Modified transfer learning module (ResNet34, VGG11) are applied to access the outcome of proposed system.
5. Every file contains performance evaluation matrix within these are summarized later on.
6. All the colab files are shared.

**Resnet34_lung_cancer**

import torch

print(torch.__version__)

# Commented out IPython magic to ensure Python compatibility.

# %matplotlib inline

import os

import numpy as np

import pandas as pd

import matplotlib.pyplot as plt

import matplotlib.patches as patches

from sklearn.metrics import roc_curve, auc

!pip install fastai

from fastai.vision.all import cnn_learner, RandTransform, ImageDataLoaders, ClassificationInterpretation, models

from fastai.metrics import error_rate, accuracy

image_directory = '/content/drive/MyDrive/LC25000/'

data = ImageDataLoaders.from_folder(image_directory, train='train', valid='val', size=224, bs=32, num_workers=8)

data.show_batch()

print(len(data.train_ds), len(data.valid_ds))

dir(models)

learn = cnn_learner(data, models.resnet34, metrics = [accuracy])

learn.summary()

learn.unfreeze()

learn.lr_find()

learn.fit_one_cycle(5,lr_max= slice(1.4e-4,1.25e-3))

learn.recorder.plot_loss(skip_start=0, with_valid=True)

learn.save('/content/drive/MyDrive/LC25000/models/lung_cancer_classifier')

interp = ClassificationInterpretation.from_learner(learn)

interp.plot_top_losses(9, figsize=(12,12))

interp.plot_confusion_matrix(figsize=(6,6), dpi=300)

interp.print_classification_report()

preds,y, loss = learn.get_preds(with_loss=True)

# get accuracy

acc = accuracy(preds, y)

print('The accuracy is {0} %.'.format(acc))

# probs from preds

probs = np.exp(preds[:,1])

# Compute ROC curve

fpr, tpr, thresholds = roc_curve(y, probs, pos_label=1)

# Compute ROC area

roc_auc = auc(fpr, tpr)

print('ROC area is {0}'.format(roc_auc))

plt.figure()

plt.plot(fpr, tpr, color='darkorange', label='ROC curve (area = %0.2f)' % roc_auc)

plt.plot([0, 1], [0, 1], color='navy', linestyle='--')

plt.xlim([-0.01, 1.0])

plt.ylim([0.0, 1.01])

plt.xlabel('False Positive Rate')

plt.ylabel('True Positive Rate')

plt.title('Receiver operating characteristic')

plt.legend(loc="lower right")

**Vgg11_lung_cancer**

# Commented out IPython magic to ensure Python compatibility.

# %matplotlib inline

import os

import numpy as np

import pandas as pd

import matplotlib.pyplot as plt

import matplotlib.patches as patches

from sklearn.metrics import roc_curve, auc

import torch

print(torch.__version__)

from google.colab import drive

drive.mount('/content/drive')

from fastai.vision.all import cnn_learner, RandTransform, ImageDataLoaders, ClassificationInterpretation, models

from fastai.metrics import error_rate, accuracy

image_directory = '/content/drive/MyDrive/LC25000/'

data = ImageDataLoaders.from_folder(image_directory, train='train', valid='val', size=224, bs=16, num_workers=8)

print(len(data.train_ds), len(data.valid_ds))

dir(models)

learn = cnn_learner(data, models.vgg11, metrics = [accuracy])

learn.summary()

learn.unfreeze()

learn.lr_find()

learn.fit_one_cycle(5,lr_max=0.00083)

learn.recorder.plot_loss(skip_start=0, with_valid=True)

learn.save('/content/drive/MyDrive/LC25000/models/lung_cancer_classifier')

interp = ClassificationInterpretation.from_learner(learn)

interp.plot_top_losses(9, figsize=(12,12))

interp.plot_confusion_matrix(figsize=(6,6), dpi=60)

interp.print_classification_report()

preds,y, loss = learn.get_preds(with_loss=True)

# get accuracy

acc = accuracy(preds, y)

print('The accuracy is {0} %.'.format(acc))

# probs from preds

probs = np.exp(preds[:,1])

# Compute ROC curve

fpr, tpr, thresholds = roc_curve(y, probs, pos_label=1)

# Compute ROC area

roc_auc = auc(fpr, tpr)

print('ROC area is {0}'.format(roc_auc))

plt.figure()

plt.plot(fpr, tpr, color='darkorange', label='ROC curve (area = %0.2f)' % roc_auc)

plt.plot([0, 1], [0, 1], color='navy', linestyle='--')

plt.xlim([-0.01, 1.0])

plt.ylim([0.0, 1.01])

plt.xlabel('False Positive Rate')

plt.ylabel('True Positive Rate')

plt.title('Receiver operating characteristic')

plt.legend(loc="lower right")
